# Supplementary material for: Stressed Actin Binding by the Prickle2 LIM Domains and its Regulation in the Full-Length Protein
Source: bioRxiv. 2025 Jun 2:2025.05.30.657073. Preprint. [Version 1] doi: 10.1101/2025.05.30.657073 (PMC12157626; doi:10.1101/2025.05.30.657073)
Supplement: 1 [file NIHPP2025.05.30.657073V1-supplement-1.pdf]

bioRxiv preprint doi: https://doi.org/10.1101/2025.05.30.657073; this version posted June 2, 2025. The copyright holder for this preprint (which was not certified by peer review) is the author/funder, who has granted bioRxiv a license to display the preprint in perpetuity. It is made available under aCC-BY 4.0 International license.

Supplemental Figure 1

A: Clustal alignment of LIM domains from *D. melanogaster* Prickle, *H. sapiens* Prickle2, and *X. laevis* Prickle2.

B: Schematic of Pk2-LCR, Pk2-LIM12, and Pk2-LIM1.

C: Pk2-LCR localizes to Zyx-LCR-labeled regions.

D-E:Pk2-LIM12 and Pk2-LIM1 fail to localize to Zyx-LCR-labeled regions.

F: Quantification of Pk2-LCR, Pk2-LIM12, or Pk2-LIM1 signal relative to Zyx-LCR.

Supplemental Figure 2

A: Predicted aligned error plot of Pk2-PET multimeric prediction with Pk2-LCR

bioRxiv preprint doi: https://doi.org/10.1101/2025.05.30.657073; this version posted June 2, 2025. The copyright holder for this preprint (which was not certified by peer review) is the author/funder, who has granted bioRxiv a license to display the preprint in perpetuity. It is made available under aCC-BY 4.0 International license.

B: Predicted aligned error plot of Pk2-PET-LCR prediction

C: Predicted aligned error plot of Pk2-LCR multimeric prediction with Pk2-Cterm

D: Predicted aligned error plot of Pk2-LCR-Cterm prediction

Supplemental Figure 3

A: Schematic of Pk2-PET, Zyx-LCR, and chimeric Pk2-PET fused to Zyx-LCR.

B: Zyx-LCR-mNeonGreen localizes to regions marked by Zyx-LCR-mScarlet3

C: Chimeric Pk2-PET-Zyx-LCR localizes to Zyx-LCR-labeled regions

D: Quantification of Zyx-LCR or Pk2-PET-Zyx-LCR signal relative to Zyx-LCR

E: Pk2-PET colocalizes with full-length Pk2

F: Pk2-Cterm colocalizes with full-length Pk2

Supplementary Video 1

TIRF microscopy of mNeonGreen-Pk2-PET, mScarlet3-Pk2, and Infrared-labeled Lifeact-HaloTag. 1 frame = 20 seconds. Scale bar, 10μm

bioRxiv preprint doi: https://doi.org/10.1101/2025.05.30.657073; this version posted June 2, 2025. The copyright holder for this preprint (which was not certified by peer review) is the author/funder, who has granted bioRxiv a license to display the preprint in perpetuity. It is made available under aCC-BY 4.0 International license.

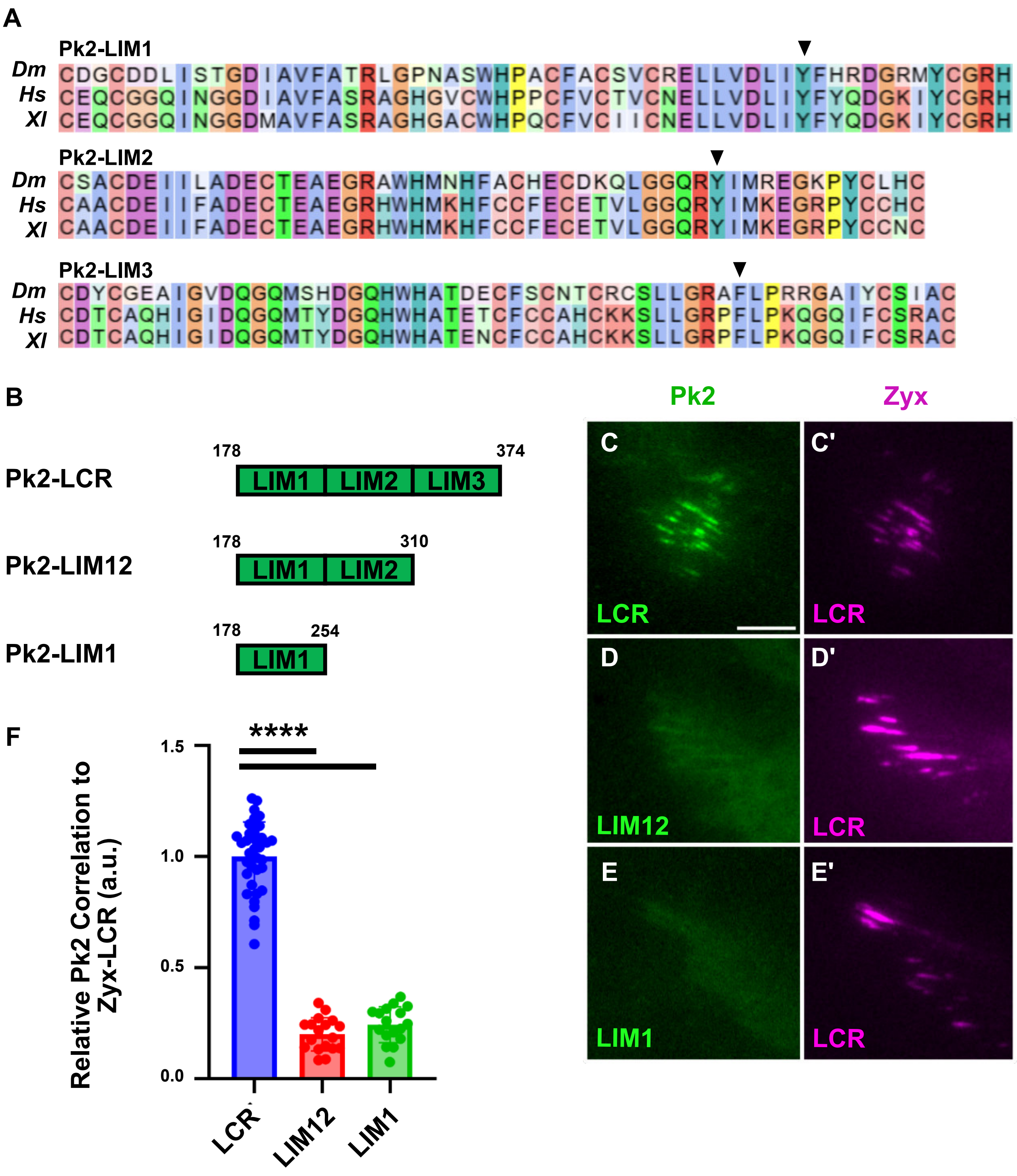

Bejar-Padilla et. al, 2025 Supplemental Figure 1

bioRxiv preprint doi: https://doi.org/10.1101/2025.05.30.657073; this version posted June 2, 2025. The copyright holder for this preprint (which was not certified by peer review) is the author/funder, who has granted bioRxiv a license to display the preprint in perpetuity. It is made available under aCC-BY 4.0 International license.

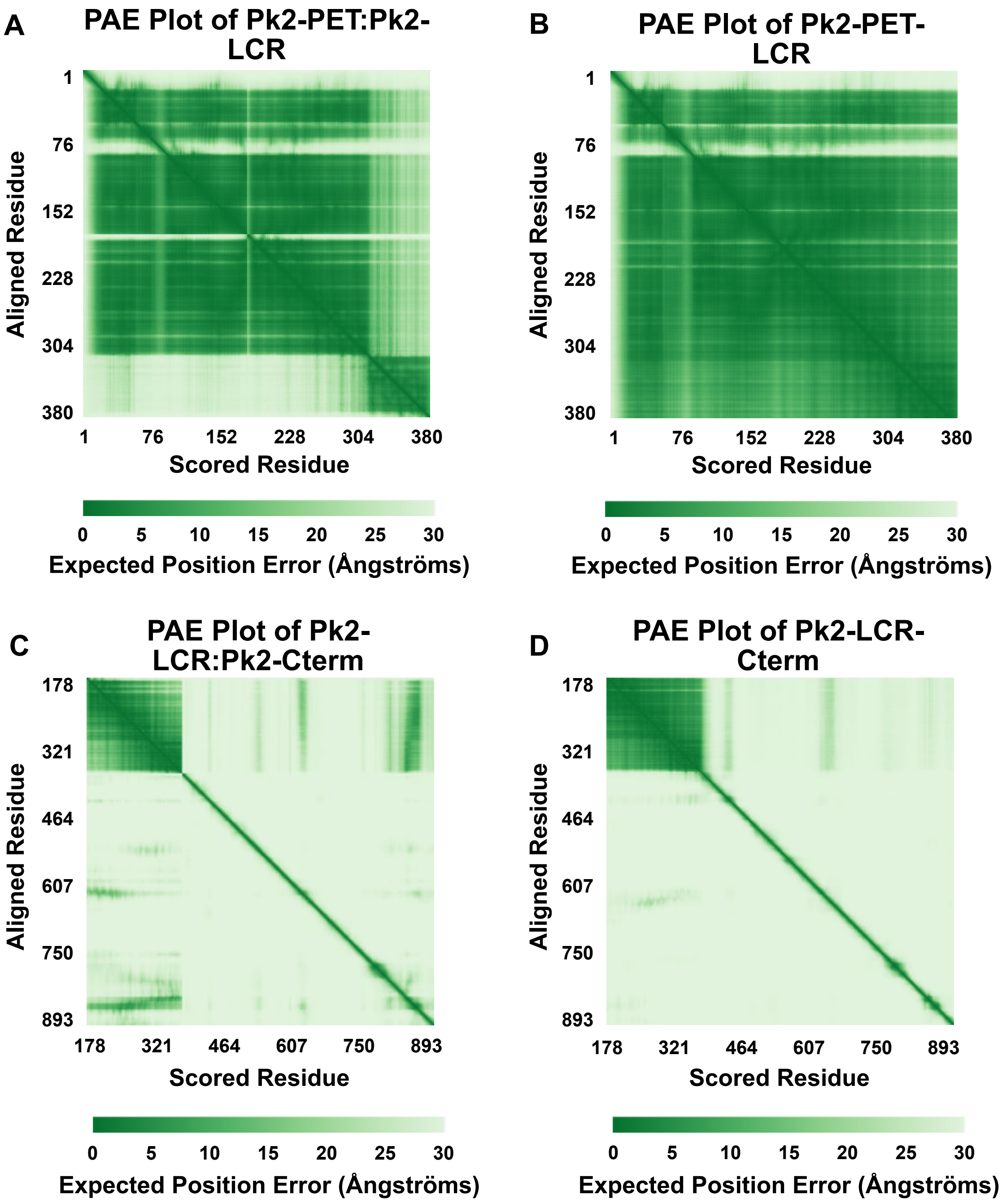

Bejar-Padilla et. al, 2025 Supplemental Figure 2

bioRxiv preprint doi: https://doi.org/10.1101/2025.05.30.657073; this version posted June 2, 2025. The copyright holder for this preprint (which was not certified by peer review) is the author/funder, who has granted bioRxiv a license to display the preprint in perpetuity. It is made available under aCC-BY 4.0 International license.

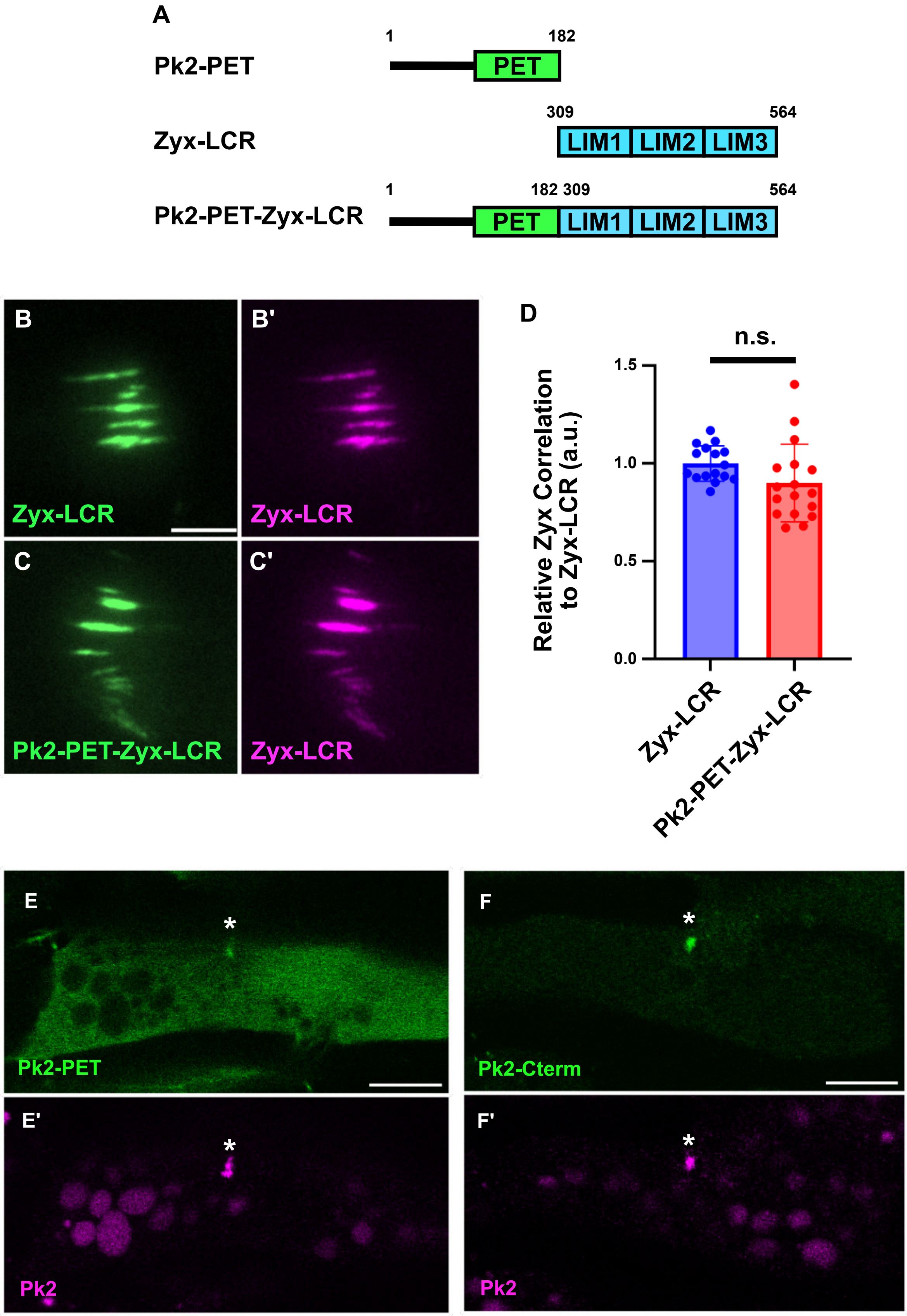

Bejar-Padilla et. al, 2025 Supplemental Figure 3
